# Application of Gene Shaving and Mixture Models to Cluster Microarray Gene Expression Data

**Published:** 2007-04-02

**Authors:** K-A. Do, G.J. McLachlan, R. Bean, S. Wen

**Affiliations:** 1 University of Texas, M.D. Anderson Cancer Center, Houston, Texas, U.S.A; 2 Department of Mathematics & Institute for Molecular Bioscience, University of Queensland Brisbane, 4072, Australia

## Abstract

Researchers are frequently faced with the analysis of microarray data of a relatively large number of genes using a small number of tissue samples. We examine the application of two statistical methods for clustering such microarray expression data: EMMIX-GENE and GeneClust. EMMIX-GENE is a mixture-model based clustering approach, designed primarily to cluster tissue samples on the basis of the genes. GeneClust is an implementation of the gene shaving methodology, motivated by research to identify distinct sets of genes for which variation in expression could be related to a biological property of the tissue samples. We illustrate the use of these two methods in the analysis of Affymetrix oligonucleotide arrays of well-known data sets from colon tissue samples with and without tumors, and of tumor tissue samples from patients with leukemia. Although the two approaches have been developed from different perspectives, the results demonstrate a clear correspondence between gene clusters produced by GeneClust and EMMIX-GENE for the colon tissue data. It is demonstrated, for the case of ribosomal proteins and smooth muscle genes in the colon data set, that both methods can classify genes into co-regulated families. It is further demonstrated that tissue types (tumor and normal) can be separated on the basis of subtle distributed patterns of genes. Application to the leukemia tissue data produces a division of tissues corresponding closely to the external classification, acute myeloid meukemia (AML) and acute lymphoblastic leukemia (ALL), for both methods. In addition, we also identify genes specific for the subgroup of ALL-Tcell samples. Overall, we find that the gene shaving method produces gene clusters at great speed; allows variable cluster sizes and can incorporate partial or full supervision; and finds clusters of genes in which the gene expression varies greatly over the tissue samples while maintaining a high level of coherence between the gene expression profiles. The intent of the EMMIX-GENE method is to cluster the tissue samples. It performs a filtering step that results in a subset of relevant genes, followed by gene clustering, and then tissue clustering, and is favorable in its accuracy of ranking the clusters produced.

## 1. Introduction

With the recent advent in DNA array technologies, researchers have recently focused on developing methods to cluster gene microarray data, and the analysis of such data has an important role to play in the discovery, validation, and understanding of various classes and subclasses of cancer; see, for example, Eisen et al. (1998); Ben-Dor et al. (1999, 2000); Alon et al. (2000); Golub et al. (2000); Hastie et al. (2000); Moler et al. (2000); and Xing and Karp (2001), among others. Most clustering procedures seek a single global re-ordering of the samples or cell lines for all genes, and although they are effective in uncovering gross global structure, they are much less effective when applied to more complex clustering patterns; for example, where there are overlapping gene clusters; see McLachlan and Basford (1988); and Xu and Wunsch (2005). In this paper, we concentrate on the gene shaving method of Hastie et al. (2000) and the mixture-model based approach of McLachlan et al. (2002), called EMMIX-GENE. EMMIX-GENE is designed primarily to cluster tissue samples on the basis of the genes. It does have an intermediate step on which the genes are clustered into groups on the basis of Euclidean distance in order to reduce the dimension of the gene space. The reader is referred to the more recent work of Heard et al. (2006); Liu et al. (2006); Ng et al. (2006); and Thalamuthu et al. (2006), among others, for model-based approaches designed specifically for the clustering of gene profiles. Gene shaving is a simple but effective method for identifying subsets of genes with coherent expression patterns and large variation across samples or conditions. To illustrate the performance of the two methods in their ability to extract true clusters, we examine the well-known data sets of Alon and Golub, while taking into account knowledge about the genes and tissue samples provided in such sources as Getz et al. (2000) and Ben-Dor et al. (2000).

## 2. Description of EMMIX-GENE

For a detailed description of the normal mixture model and the extensions to mixtures of *t* distributions and mixtures of factor analyzers, see McLachlan et al. (2002). Very briefly, we let α_1_, …, α*_N_*, denote *N p-*dimensional observations. We then attempt to use the expectation-maximization (EM) algorithm of Dempster et al. (1977) to assign the observations to *g* different normal components.

The EMMIX-GENE program uses a three-part process: first, the selection of relevant genes; second, the clustering of the selected genes; and third, the clustering of the tissues of the basis of the selected genes or gene clusters. At each step, the EMMIX program of McLachlan et al. (1999) is used to fit mixtures of normal components or *t* components to the data. The analysis is to be performed on microarray data collected on *N* genes from *p* experiments, represented in the form of an *N* × *p* data matrix *A* whose *i*th row contains the expression levels for the *i*th gene in the *p* tissue samples.

### 2.1. Selection of relevant genes

In the first step, consideration is given to the selection of relevant genes in terms of the likelihood ratio statistic −2 log λ for the fitting of a single *t* distribution versus a mixture of two *t* components. Due to the possible presence of atypically large expression values for a particular tissue in the microarray data, it is better to use mixtures of *t* components as opposed to mixtures of normal components. When assessing the relevance of a gene, we examine −2 log λ where λ is the likelihood ratio statistic for testing *g* = 1 versus *g* = 2 components in the mixture model.

However, the use of *t* components in place of normal components still does not eliminate the effect of outliers on inference of the number of clusters in the tissue samples. For example, suppose that for a given gene there is no genuine clustering in the tissues, but that there are a small number of gross outliers. Then a significantly large value of λ might be obtained, with one component representing the main body of the data (and providing robust estimates of their underlying distribution) and the other representing the outliers. That is, although the *t* mixture model may provide robust estimates of the underlying distribution, it does not provide a robust assessment of the number of clusters in the data.

In light of the above, the EMMIX-GENE software automatically assesses the relevance of each of the *N* genes by fitting one- and two-component *t* mixture models to the expression data over the *p* tissues for each gene considered individually. If −2 log λ is greater than a specified threshold *b*_1_

(1) $$-2\;\text{log}\;\lambda \;>\;b_{1}$$

then the gene is taken to be relevant provided that

(2) $${\text{s}}_{\text{min}}\;\geq \;b_{2},$$

where s_min_ is the minimum size of the two clusters implied by the two-component *t* mixture model and *b*_2_ is a specified threshold.

**Figure 1.**
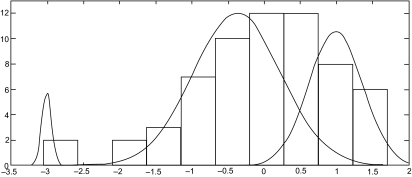
Histogram of Gene 474 (T70046) with Mixture of *g* = 3 Fitted *t* Components.

If (1) holds but (2) does not for a given gene, then the three-component *t* mixture model is fitted to the tissue samples on this gene, and the value of −2 log λ calculated for the test of *g* = 2 versus *g* = 3; see Figure 1. If (1) holds for this value of −2 log λ the gene is selected as being relevant. Although the null distribution of −2 log λ for *g* = 2 versus *g* = 3 is not the same as for *g* = 1 versus *g* = 2 components, it would appear to be reasonable here to use the same threshold (1).

For the data discussed in this paper, we took *b*_1_ = 8 and *b*_2_ = 8. In fitting the two- and three-component *t* mixture models to the tissue samples, we need to provide a starting point for the parameter estimate, or equivalently, the clustering of the data. This can be done by the user specifying a number of random starts and a number of *k*-means-based starts. In EMMIX-GENE, the default choice is four random and four *k*-means-based starts, which is used in the analyses presented later.

This first step is responsible for most of the time taken to implement EMMIX-GENE. If a less formal method of selection of the genes were to be used, then the procedure would be very quick. For example, one ad hoc method for selecting the genes would be to select those genes whose sample interquartile range is greater than some specified multiplicative factor of the sample standard deviation.

### 2.2. Clustering of genes

Concerning the end problem of clustering the tissue samples on the basis of the genes considered simultaneously, we could examine the univariate clusterings provided by each of the selected genes taken individually. But this would be rather tedious when a large number of genes have been selected. Thus, with the EMMIX approach, the genes are clustered into a user-specified number (*N*_0_) of clusters by fitting a mixture of g = *N*_0_ normal distributions with covariance matrices restricted to being equal to a multiple of the *p* × *p* identity matrix. In particular, if the mixing proportions were fixed at 0.5, then it would be equivalent to using *k*-means and clustering the genes in terms of Euclidean distance between them. One could attempt to make a more objective choice of the number *N*_0_ of clusters by using, say, the likelihood ratio criterion or the Bayesian information criterion (BIC), but regularity conditions do not hold for this problem. Moreover, there is an extra complication here since the genes are not independently distributed within a tissue sample. The clusters of genes are ranked in terms of the likelihood ratio statistic calculated on the basis of the fitted mean of a cluster over the tissues for the test of a single versus two *t*-distributions. If the smaller cluster is found to be of size less than *b*_2_, the test is run for two versus three *t*-distributions.

### 2.3. Clustering of tissues

In the last step, the tissues are clustered by fitting mixtures of factor analyzers to the genes where the information in all the genes has been condensed as above. Factor analysis can be used for dimensionality reduction by modeling each observation *A_j_* as

(3) $$\alpha_{j}=\mu +BU_{j}+e_{j}\;(j=1,\;\ldots ,\;n),$$

where *U****j*** is a *q*-dimensional (*q* < *p*) vector of latent or unobservable variables called factors and *B* is a *p* × *q* matrix of factor loadings (parameters). The *U****j*** are assumed to be independent and identically (i.i.d.) as *N* (0, *I* *_q_*), independently of the errors *e_j_*, which are assumed to be i.i.d. as *N*(0, *D*), where *D* is a diagonal matrix,

$$D=\text{diag}(\sigma_{1}^{2},\ldots \sigma_{\text{p}}^{2}),$$

and where *I_q_* denotes the *q* × *q* identity matrix. Thus, conditional on the *u_j_*, the *a_j_* are independently distributed as *N*(μ + *B u_j_*, *D*). Unconditionally, the *a*_j_ are i.i.d. according to a normal distribution with mean μ and covariance matrix

(4) $$\sum =BB^{\text{T}}+D.$$

With model (3), we avoid having to compute the inverses of iterates of the estimated *p* × *p* covariance matrix ∑ that may be singular for large *p* relative to *n*. The reason for this is that at each iteration the inversion of the current value of the *p* × *p* matrix (*BB*^T^ + *D*) can be undertaken using only inverses of *q* × *q* matrices. See McLachlan et al. (2002) for more details.

## 3. Description of the Gene Shaving Algorithm

*Gene shaving* (Hastie et al. 2001) searches for clusters of genes showing both high variation across the samples, and correlation across the genes. Both of these important aspects cannot be captured by simple clustering of the genes, or thresholding of individual genes based on the variation over samples (such as a simple *t* –test or rank test). The gene shaving procedure involves an iterative algorithm based on the principal components or the singular value decomposition (SVD) of the data matrix. The algorithm starts with the *N* × *p* matrix *A* (the entire microarray gene expression dataset) and seeks a gene cluster in the direction of maximal variation across the tissue samples. The simplest form of this function is a normalized linear combination of the genes weighted by its largest principal component loadings, referred to as the *super gene*. The genes may be sorted according to the principal component weights. A fraction α of the genes having lowest correlation (essentially the absolute inner product) with the *super gene* are then *shaved* off (discarded) from the original data matrix. The process of calculating the leading principal component and shaving off some genes is iterated on the updated/reduced data matrix until only two genes remain, which generates a nested sequence of clusters *A* = *B*_0_ ⊃ *B*_1_ ⊃ *B*_2_ ⊃ … ⊃ *B*_s._ The method thus requires a quality measure for an optimal cluster. A *Gap* function was used by Hastie et al. (2001) to select a reasonable cluster size from the sequence of nested clusters. For a particular gene block *B_s_* of *k* rows with elements α*_ij_*, define the *percent variance explained* as

$$R^{2}(B_{S})=100\times \frac{V_{B}}{V_{B}+V_{w}}==100\times \frac{V_{B}/V_{w}}{1+V_{B}/V_{w}}$$

where *V_W_*, *V_B_* and *V_T_* denote the within, between, and total variances for cluster *B_s_*. The range of *R*^2^ is over the interval [0, 1] where values close to 0 imply no clustering evidence, while values closer to one imply tight clusters of similarly expressing genes.

Let *NumPerm* be the pre-defined number of permutations, and A^*1^, ..., A^*^ *^NumPerm^* denote the corresponding permuted data matrices, obtained by permuting the elements within each row of *A*. For each generic A^*^, we form a nested sequence of clusters $A^{*}=B_{0}^{*}⊃B_{1}^{*}⊃B_{2}^{*}⊃\ldots ⊃\;B_{s}^{*}$ with corresponding cluster sizes *N* = k_0_, k_1_, ..., k*_s_* = 2 respectively. Under the null hypothesis of independence between genes (rows) and samples (columns) ${\bar{R}}_{k_{s}}^{2*}$ denote the averaged estimated value of the percent variance explained by clusters of size, computed from *NumPerm* permutations. The *Gap function* for cluster *B_s_* of size *k_s_* is defined as

(5) $$\mathrm{Gap}(k_{s})=R_{k_{S}}^{2}(B_{S})-{\bar{R}}_{k_{S}}^{2*}.$$

The optimal cluster size is the value *k_opt_* that maximizes the Gap statistic over all values of *k_s_* ∈ {2,3, ..., *N*}. Implementation of the Gap statistic criterion is enhanced by plotting the percent variance curve $R_{k_{s}}^{2}(B_{s})$ for the observed data matrix, versus the corresponding averaged curve ${\bar{R}}_{k_{s}}^{2*}$ for the collection n of permuted data matrices, as a function of *k_s_* ∈ {2, ..., *N*}. Alternatively, one can also include a plot of the computed values of Gap(*k_s_*) against *k_s_*∈ {2, ..., *N*}. Since the optimal cluster size usually assumes a small integer, these plots are more meaningful if depicted on the log scale.

The next step is to remove the effect of genes in the optimal cluster, *C*_1_ say, from the original matrix *A*. By computing the average gene or the vector of column averages for *C*_1_, denoted by *C̄*_1_, we can remove the component that is correlated with this average. This is equivalent to regressing each row of *A* on the average gene row *C̄*_1_, and replacing the former with the regression residuals. Such a process was referred to as *orthogonalization* by Hastie et al. (2001), from which a modified data matrix *A*_ortho_ is produced. The next optimal cluster C_2_ then can be obtained by using the same procedure with the data matrix *A*_ortho_, a substitution of *A*. This sequence of operations is iterated until *M* gene clusters *C*_1_, ..., *C_M_* are found, which can be displayed graphically for visual inspection. To allow for negatively correlated genes to be included in a cluster, the average gene is actually a *signed mean gene*, that is a gene row has a negative principal component weight, and then the signs of the expression values are flipped before the average is calculated.

A fully *supervised shaving* for class discrimination can be carried out if information of the column (sample) classification is available. In particular, suppose that the *p* columns (samples) can be classified by *g* groups, labeled by *G*_1_, ..., *G_g_* with *n*_1_, ..., *n_g_* columns in each group, define a *p* × *g* matrix

$$Q^{T}=\left(\begin{array}{l} \frac{1}{\sqrt{n_{1}}}\;\ldots \;\frac{1}{\sqrt{n_{1}}}\;0\;\ldots \;0\;\ldots \;0\ldots 0\\ 0\;\ldots \;0\;\frac{1}{\sqrt{n_{2}}}\;\ldots \;\frac{1}{\sqrt{n_{2}}}\;\ldots \;0\;\ldots \;0\\ \ldots \\ 0\;\ldots \;0\;\;0\;\ldots \;0\;\ldots \;\frac{1}{\sqrt{n_{g}}}\;\ldots \frac{1}{\sqrt{n_{g}}} \end{array}\right)$$

It can be easily proven that the cluster *C_k_* of rows of the data matrix *A* with maximal *between-group variance* (with respect to its mean gene) is also the cluster that maximizes the sum of squares of the mean of the rows of *A*^†^ = *AQ*, a matrix with fewer columns than *A*.

The difference between supervised and unsupervised shaving algorithms is that, in order to generate the nested sequence of clusters *B*_0_ ⊃ *B*_1_ ⊃ *B*_2_ ⊃... ⊃ *B_S_*, the unsupervised shaving algorithm calculates the principal component of *A*, while the supervised shaving algorithm calculates the principal component of *A*^†^. Note that each column of *A*^†^ is a weighted linear combination of the columns of A. In particular, the weight is equal to $\frac{1}{\sqrt{n_{i}}}$ if the *i*^th^ column belongs to group *G_i_* and zero if otherwise. Hence each column of *A*^†^ is the ‘representative’ of the group of columns (samples) of A; thus, supervised shaving maximizes a weighted combination of column variance. Another advantage is that the computing speed of supervised shaving is faster than unsupervised shaving since *A*^†^ has much fewer columns than *A*. In addition, the method can be developed to more general situation. For example, the amount of supervision can be modified to get a partially supervised algorithm (see Hastie et al. 2001). Specifically, a partially supervised gene shaving algorithm with prior external classification knowledge is based on maximizing a weighted combination of the column means variance and an information measure that is the sum of squared differences between the class averages. That is, the original matrix *A* is first transformed by the projection P (*A*) = A* = A*Q* where

(6) $$Q*\;{Q*}^{T}=(1-\omega )\;I+\omega Q*Q^{\text{T}}\;\omega \in [0,1],$$

and then gene shaving is performed on the transformed matrix A*. Full supervision is equivalent to ω = 1; while partial supervision is indicated by values of ω between 0 and 1.

When the external information of the *p* samples is in the form of a continuous variable *Y*, then one can define the quality measure for a cluster mean by the strength of its regression on *Y.* For example, consider the case when survival times *Y* for the samples are observed, then the relationship of *Y* and *A* may be represented by a Cox proportional hazards model of *Y* to the covariate represented by the column averages of *A* via the coefficient β, where β = 0 indicates no relationship. Let the vector of score components evaluated at β = 0 be represented by a *p* × 1 vector *s* with components *s_j_*(0) for *j* = 1, ..., *p*. Under this scenario, the projection of *A* is of the form *P*(*A*) = A* = A* Q* where

(7) $$Q*\;{Q*}^{T}=(1-\omega )\;I+\omega \text{s}\;*\;{\text{s}}^{\text{T}}\;\omega \in [0,1].$$

Thus under full supervision, gene shaving is equivalent to simply ranking the genes in order of the size of the Cox model score test.

In many applications, we note that the Gap curve of the gene shaving clusters may be flat near the maximum, or may not be unimodal. This implies that there are larger cluster sizes that may include additional genes highly correlated with the super gene in this specific cluster with a Gap statistic almost as large as the Maximum Gap value. An automatic implementation of choosing the cluster size according to the Maximum Gap statistic usually would end up with smaller cluster sizes than other methods, but with much higher coherence in the cluster. We devised a simple extension to the original gene shaving algorithm, by allowing the user to relax the Maximum Gap Statistic criterion, that is, the user can pick the cluster size within a certain percentage (say 5% or 10%) of the Maximum Gap Statistic. Perhaps an improved version of cluster size selection should be based on both the modulus and the slope of change of the Gap statistic with respect to cluster size. This requires further investigation and is beyond the scope of this paper.

The gene shaving algorithm under general supervision with the Gap Statistic relaxation option has been implemented (and is continuously updated) by our group in the Department of Biostatistics at M. D. Anderson Cancer Center. There are two versions:

- An implementation (entirely in S and R) of gene shaving (including unsupervised and general supervision) and documentation can be downloaded from http://lib.stat.cmu.edu/S/
- GeneClust (http://odin.mdacc.tmc.edu/~kim/gene-clust): is a suite of Splus/R functions and C routines with a graphical user interface written in JAVA. This allows the user the ability to interact directly with the program, to have visualization power of the data and resulting clusters, and to have control of numerous intermediate output results.

In the next section, we present an illustration of how the two software GeneClust and EMMIX-GENE can be applied to the analysis of two widely studied cancer data sets. Note that for the sake of simplicity, we use the generic term “gene ID” here to refer to “Affymetrix Probe Set ID” for data collected from Affymetrix oligonucleotide arrays.

## 4. Colon Data

Alon et al. (1999) used Affymetrix oligonucleotide arrays to monitor absolute measurements on expressions of over 6,500 human gene expressions in 40 tumor and 22 normal colon tissue samples. These samples were taken from 40 different patients so that 22 patients supplied both a tumor and normal tissue sample. Alon et al. (1999) focused on the 2,000 genes with highest minimal intensity across the samples, and it is these 2,000 genes that comprise our data set. The microarray data matrix *A* for this set thus has *N* = 2,000 rows and *M* = 62 columns. In Alon et al. (1999), the tissues are not listed consecutively, but here we have rearranged the data so that the tumors are labeled 1 to 40 and the normals 41 to 62. Also, since several of the Affymetrix probe sets have the same IDs, we differentiate between these IDs by the addition of an underscore and a number. We use the generic term “gene” to refer to a “probe set” in this case. Thus, for example, the first occurrence of gene H43908 in the list of 2000 genes is still called H43908, but the two subsequent occurrences are labeled H43908_2 and H43908_3.

Getz et al. (2000) reported that there was a change in the protocol during the conduct of the microarray experiments. The 11 tumor tissue samples (labeled 1 to 11 here) and 11 normal tissue samples (41 to 51) were taken from the first 11 patients using a poly detector, while the 29 tumor tissue samples (12 to 40) and normal tissue samples (52 to 62) were taken from the remaining 29 patients using total extraction of RNA. In the following, we find some evidence of this change in protocols in the clusterings we discovered.

Before we considered the clustering of this set, we processed the data by taking the (natural) logarithm of each expression level in *A*. We subsequently normalized the columns of the microarray data to have mean zero and unit standard deviation, then standardized the rows of the resulting matrix to have mean zero and unit standard deviation.

### 4.1. EMMIX-GENE approach

The gene selection approach of Section 2.1 was applied, with thresholds of *b*_1_ = *b*_2_ = 8, which retained 446 genes from the original 2,000 genes. The 446 selected genes were split into twenty clusters by fitting a mixture of twenty normal distributions with covariance matrices restricted to being equal to a multiple of the 62 × 62 identity matrix. These twenty clusters ranged in size from 8 to 41. In McLachlan et al. (2002) it was noted that the second Alon cluster as produced by EMMIX-GENE gave a clustering of the tissues *C*_2_ as follows.

$$\begin{array}{l} C_{2}=\{1-29,\;31-32,\;34-35,\;37-40,\;48,\;58,\;60\}\\ \;\;\;\;\;\;\;\;\;\;\;\cup \{30,\;33,\;36,\;41-47,\;49-57,\;59,\;61-62\}. \end{array}$$

We note that the error rate of *C*_2_ compared with the external classification is six, the lowest of any of the twenty clusters, and that this cluster of genes contains the smooth muscle genes and genes suspected of being related to smooth muscle that were mentioned in Ben-Dor et al. (2000). (Ben-Dor et al. noted that the normal colon biopsy included smooth muscle tissue from the colon walls and consequently smooth muscle-related genes showed high expression levels in the normal tissues samples compared to the tumor samples.) However, it should be noted that the six tissues which are misallocated under this clustering occur among those tissues which have been misallocated in other analyses of this data set. For example, with the support vector machine classifier formed in Moler et al. (2000) using the known classification of the tissues, these six tissues along with tumor tissue 35 were misallocated in the (leave-one-out) cross-validation of this classifier. Thus the “true” classification of these six tissues is in doubt. On comparing the clustering C_2_ and the true classification, the Rand index was found to be 0.82.

**Figure 2.**
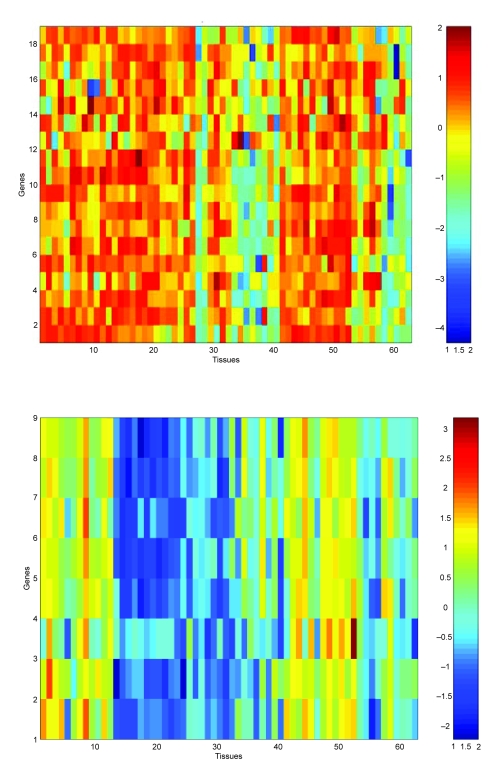
Heat maps of EMMIX-GENE clusters for the colon data.

In Figure 2, we show two heat maps extracted from fitting twenty clusters using EMMIX-GENE. We also note that all the smooth muscle related genes and suspected smooth muscle related genes mentioned in Ben-Dor et al. (2000) are placed in C_2_ represented by the upper heat map in Figure 2. The lower heat map corresponds essentially to a dichotomy between tissues obtained under the “old” and “new” protocols, note the similarity of columns 1–11 and columns 41–51, representing the “old” protocol. On a Pentium-4 with a 3.2 GHz processor, the gene selection step takes 87 minutes for the full set of 2000 genes; the gene clustering step takes 85 seconds to cluster the reduced set of 446 genes into twenty clusters; and the tissue clustering step takes two seconds working with the mean of one of the clusters.

### 4.2. Gene shaving approach

**Figure 3.**
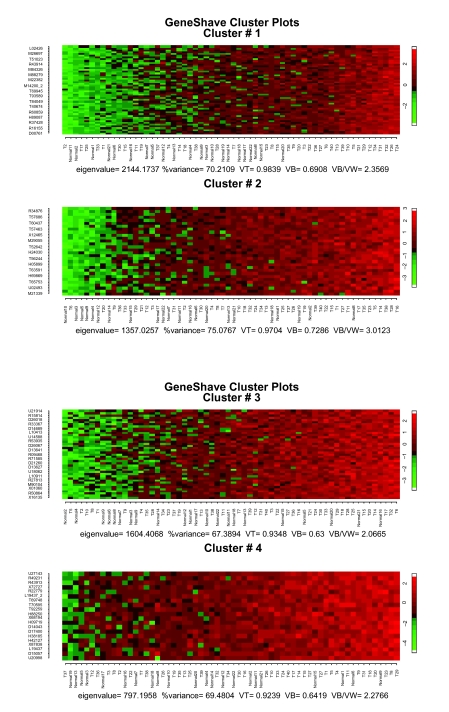
Heat maps of the first four unsupervised gene shaving clusters for the colon data, sorted by the column mean gene.

**Figure 4.**
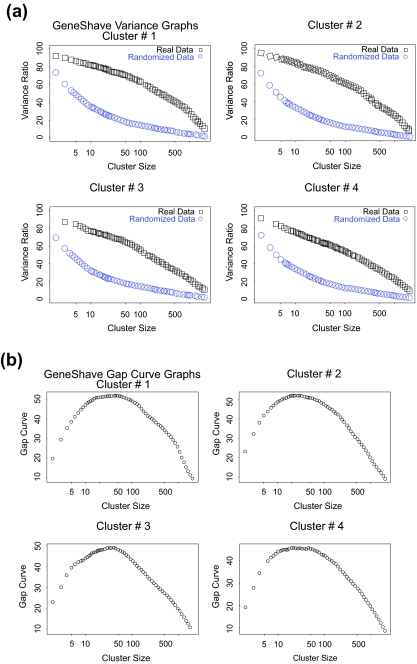
(*a*) Variance plots for the original and randomized data. The percent variance explained by each cluster, both for the original data, and for an average over twenty randomized versions. (*b*) Gap estimates of cluster size. The Gap curve corresponds to the difference between the pair of variance curves.

The gene shaving algorithm was then applied to the same data set of 2000 genes used by the EMMIX-GENE approach. On a Pentium-4 with a 3.2 GHz processor, the overall gene shaving procedure takes less than 2 minutes to extract 4 clusters and using 20 permutations per cluster. Heat maps of the first four gene-shaved clusters using 10% shaving and 20 permutations are presented in Figure 3. Figure 4 shows the percent-variance curves for both the original and randomized data as a function of size, and the gap curves used to select the specific cluster sizes in Figure 3. Visual examination of the first four unsupervised gene-shaved clusters reveals some interesting patterns. The first cluster of 50 genes group 25 of the tumors to the right, indicating that these specific genes are highly expressed in tumors. The second cluster of 40 genes can be interpreted similarly, though the pattern of high expression is different from that of the first cluster. The third cluster of 41 genes corresponds to the clustering of the “old” versus “new” protocols where most samples (tumor and normal) from the 11 patients using a poly detector are mostly under expressed for these genes and are grouped towards the left hand side of the heat map. Subsequent clusters display coherent patterns of expression with high values of *V_B_* */ V_W_* but do not suggest any clear clusterings that resemble either the external classification or the change in protocol paradigms identified by Getz (2000).

**Figure 5.**
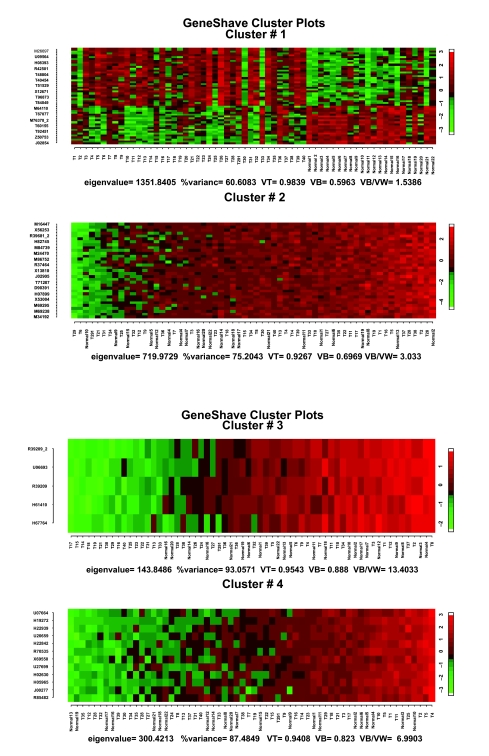
Heat maps of the first four gene shaving clusters for the colon data with 50% supervision. The samples for clusters 2–4 are sorted by the column mean gene.

We also reanalyzed the full Alon data set with different levels of supervision ranging from 10% to 100% supervision using the external classification of tumor versus normal. With 50% supervision, the first four gene clusters are presented in Figure 5. The first cluster (samples are not reordered) shows 50 genes (including the two smooth-muscle genes J02854 and T60155) representing two distinct groups of negatively correlated genes that correspond well to the external classification. The third cluster of 5 genes (sorted by the column means of the cluster) group the tissues according to the old versus new protocols. When 100% supervision is used (Figure 6), the most coherent cluster that correspond to the external classification consists of 9 genes and classifies the tumors and normals with an error rate of 6 (Rand index of 0.82), as found by other methods. These nine genes also correspond to those with the top TNoM scores used by Ben-Dor et al. (2000). TnoM is the threshold number of misclassification which measures the “relevance” of a gene. Inspection of the variance and Gap plots under the full supervised scenario indicates that only the first cluster captures the full external classification.

We relaxed the Maximum Gap Statistic to pick the largest cluster size within 5% of the Maximum Gap value. Under 50% supervision, the relaxed gene shaving method identifies the first gene-shave cluster with 77 genes, capturing the normal versus tumor structure and including all the six smooth muscle genes (J02854, T60155, M63391, D31885, X74295, X12369) as well as two ribosomal genes (T95018, T62947).

**Figure 7.**
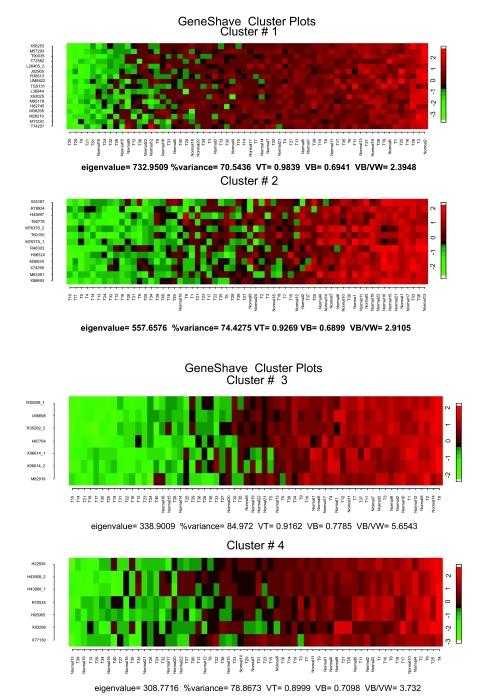
Heat maps of the first four unsupervised gene shaving clusters for the reduced colon data (446 genes), sorted by the column mean gene.

We further investigated the performance of unsupervised gene shaving when applied to the reduced Alon data set of 446 genes using a preliminary filtering step similar to the first step in EMMIX-GENE. The first four gene shave clusters are presented in Figure 7. It can be seen that cluster 2 (13 genes) captures the normal versus tumor structure quite well, while the change in paradigm structure is reflected in cluster 3 (7 genes). Application of the relaxed gene shaving method increases cluster 2 to 19 genes and cluster 3 to 17 genes while maintaining the discovered structures. Further, cluster 2 now includes all the smooth muscle genes.

## 5. Leukemia Data

Golub et al. (1999), studied gene expressions on two types of acute leukemia: acute lymphoblastic leukemia (ALL) and acute myeloid leukemia (AML). Gene expression levels were measured using Affymetrix high density oligonucleotide arrays containing *N* = 7,129 genes on *M* = 72 tissues, comprising 47 cases of ALL (38 B-cell and 9 T-cell ALL) and 25 cases of AML. We have rearranged the order of the tissues so that the first 47 columns of the microarray data matrix *A* refer to the ALL cases and the next 25 to the AML cases.

We followed the processing steps of Dudoit et al. (2002) of (i) thresholding: floor of 100 and ceiling of 16,000; (ii) filtering: exclusion of genes with max/min ≤ 5 and (max–min) ≤ 500, where max and min refer respectively to the maximum and minimum expression levels of a particular gene across a tissue sample; (iii) the natural logarithm of the expression levels was taken (Dudoit et al. (2002) used base 10 logarithms). This left us with 3,731 genes.

### 5.1. EMMIX-GENE approach

Firstly, the approach of Section 2.1 was used, retaining 2,015 genes from the original 3,731 genes. Using the same approach as was applied to the Alon data, we clustered the 2,015 genes into forty clusters using the EMMIX-GENE program. The sizes of the forty clusters ranged from 14 to 137. We observe that the EMMIX-GENE approach gives an error rate of nine when the first or third cluster from the forty Golub clusters was used to cluster tissues using a mixture of factor analyzers. The clustering is *C*_1_ for the first cluster and *C*_3_ for the third cluster as follows:

$$\begin{array}{l} C_{1}=\{1-4,\;8-43,\;45-47,\;49,\;52-53,\;67,\;69\}\\ \;\;\;\;\;\;\;\;\;\;\;\cup \;\{5-7,\;44,\;48,\;50,\;51,\;54-66,\;68,\;70-72\}.\\ C_{3}=\{1-10,\;13,\;15-16,\;19-22,\;24-28,\;30,\;32-43,\;45-47\}\\ \;\;\;\;\;\;\;\;\;\;\cup \;\{11-12,\;14,\;17-18,\;23,\;29,\;31,\;44,\;48-72\}. \end{array}$$

Thus, these clusters correspond closely to the external classification of ALL versus AML tumors. By examining the heat maps, we can see that this pattern is evident for the forty clusters in general. We measured the Rand index for both of these clusters and they both equal 0.78.

**Figure 8.**
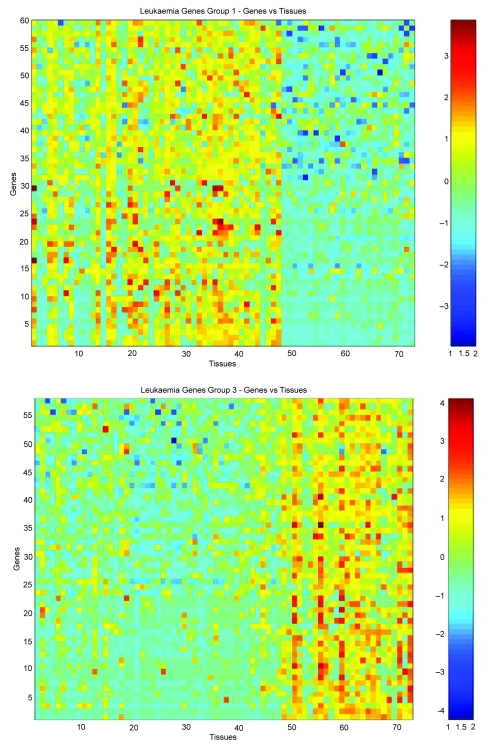
Analysis of leukemia data by EMMIX-GENE. Heat maps of two main clusters from fitting 40 clusters.

We present the heat maps for the two representative EMMIX-GENE clusters (extracted from forty clusters) in Figure 8. Using the second gene cluster as a basis for clustering the 72 tissues produces the best results; for example, using a mixture *q* = 6 factor analyzers gives a clustering in which only six tissues are misallocated (44, 49, 52, 53, 67 and 69). If the group mean of this cluster is used, we arrive at a clustering in which only five tissues are misallocated (44, 49, 52, 53 and 69).

### 5.2. Gene shaving approach

**Figure 9.**
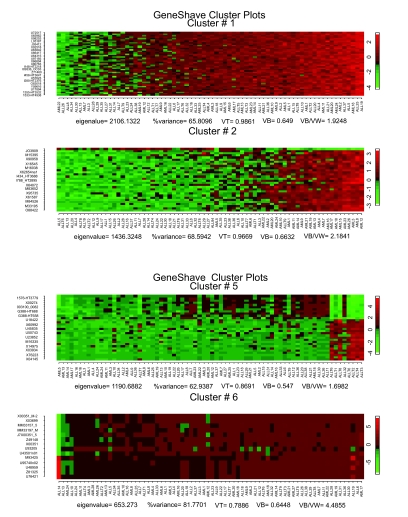
Heat maps of four of the first six unsupervised gene shaving clusters for the leukemia data, sorted by the column mean gene.

**Figure 10.**
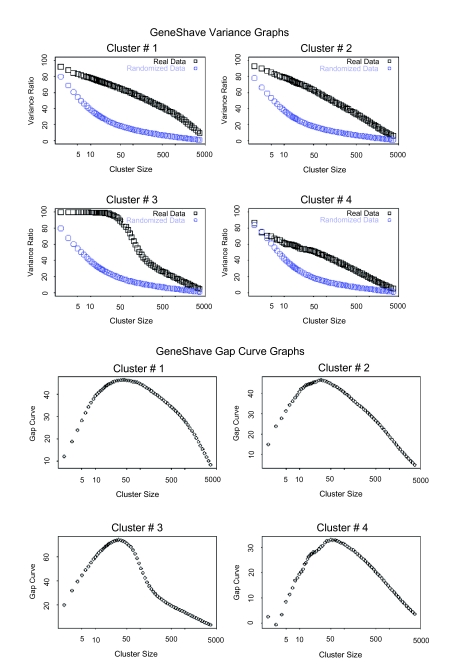
(**a**) Variance plots for the original and randomized data. The percent variance explained by each cluster, both for the original data, and for an average over twenty randomized versions. (**b**) Gap estimates of cluster size. The Gap curve corresponds to the difference between the pair of variance curves.

We first applied unsupervised gene shaving to the original 3,731 genes in the Golub data set, using 10% shaving and 20 permutations to calculate the Gap statistics. Four of the top six clusters are presented in Figure 9. The variance and Gap plots for the first four clusters are depicted in Figure 10. Cluster 2 consisting of 30 genes depicts a clear grouping of the AML versus ALL tumor types, with a Rand Index of 0.78. The genes that are specifically differentially expressed for the ALL-Tcells alone are captured by the fifth cluster (30 genes) which shows two subgroups of positively and negatively correlated genes (Rand Index = 1.0). Due to the orthogonality property of gene shaving, other clusters may show high coherence amongst the genes but are not expected to adhere to the structures already captured by clusters 2 and 5.

**Figure 11.**
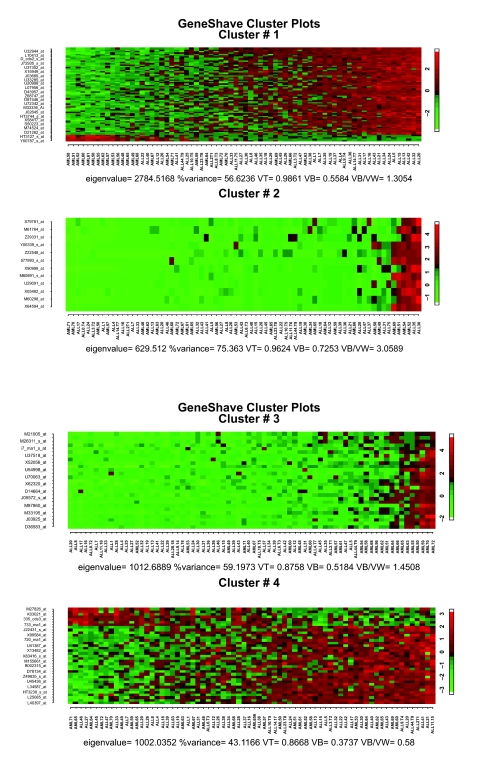
Analysis of the reduced Golub data set (2,015 genes). (**a**) Heat maps of the first four unsupervised gene shaving clusters; the samples are sorted by the column mean gene.

We also applied the relaxed unsupervised gene shaving approach to the reduced Golub data set of 2,015 genes after the preliminary filtering. The resulting top four gene-shave clusters are given in Figure 11. With this reduced data set, the structure of AML versus ALL tumor types is captured immediately in the first gene-shave cluster of 69 coherent genes (Rand-index = 0.78). Specific differentially expressing genes for the ALL-Tcells are captured by the third cluster of 27 genes (Rand-Index = 1.0).

We observe that the EMMIX-GENE clusters which produced *C*_1_ and *C*_3_ above each contains many genes with very high values of −2 log λ. For example, the first EMMIX-GENE cluster contains eight genes (out of fifty-nine) with −2 log λ values above the highest value found in the gene shaving clusters, 62.870. The third EMMIX-GENE cluster also contains eight genes above this value.

There is a clear correspondence between the clusters produced by the gene shaving method and those produced by EMMIX-GENE. The highest value of −2 log λ for the selected genes in the Alon data is 238.770. In the gene shaving clusters, the highest value of −2 log λ for any gene is 169.954. This is the second gene (out of 446) in descending order of −2 log λ. For the Golub data, the highest value of −2 log λ is 133.394, and the highest in the gene shaving clusters is 62.870. This is the sixty-third gene, out of 2,015, in descending order of −2 log λ .

If we take each pair of genes in each gene cluster, we may calculate the correlation coefficient between such pairs of genes. For the gene shaving clusters, the coefficient is between 0.39 and 1.00 for the top three Golub clusters, and between 0.8581 and 0.9994 in the case of the third cluster. Some negative correlation occurs in all these clusters except for numbers 1, 3 and 7. Considering the coefficients for the Alon clusters, we find that these are always positive and lie between 0.157 and 0.974. If we omit cluster 7, the coefficients lie between 0.332 and 0.925.

For the Alon clusters, such correlation does not exist in the larger EMMIX-GENE clusters; for example, for the 59 genes in the first Golub cluster, the correlation coefficient ranges from 0.01 to 0.88. Taking the top 10 by 2 log λ from this cluster changes this range to 0.41 to 0.88, and taking the top five gives 0.65 to 0.88. In general, for EMMIX-GENE clusters, the top genes in terms of −2 log λ are more highly correlated than the remaining genes.

We observe that four of the gene shaving clusters in the case of the Alon colon cancer data are subsets of exactly one EMMIX-GENE cluster, and three clusters are subsets of exactly one EMMIX-GENE cluster in the case of the Golub data. Focussing on the full Alon cancer data set, we find that the smooth muscle genes (J02854, T60155, M63391, D31885, X74295, X12369), each has estimated empirical Bayes posterior probability of differential expression that is greater than **0.95**. These genes are found within the top 66 ranked genes using EMMIX-GENE and within the top 78 ranked genes using t-test with a false discovery rate (FDR) of 0.001. For this data set, Do et al. (2005) conducted a full Bayesian mixture modeling approach and estimated the posterior probability of differential expression for each smooth muscle gene to be greater than 0.998. In their original analysis, Alon et al. (1999) identified a ribosomal gene cluster (29 genes), associated with over-expression in tumor tissues relative to normal tissues, only 10 which are declared differentially expressed at a threshold of 0.95 posterior probability using EMMIX-GENE, supported by similar results in Do et al. (2005) and by standard t-test with an FDR correction.

## 6. Discussion

In general, there is no obvious way to compare the clusters obtained between the two methods since the basic criterion of cluster choice is quite different. Extensive surveys of clustering algorithms including rigorous comparisons have been addressed in the literature, see for example, Yeung et al. (2001); Dudoit et al. (2002); Gibbons and Roth (2002); Costa et al. (2004); Xu (2005). Our main aim of this article is to demonstrate the usefulness of two recent methodology along with the available software in the analysis of two publicly available data sets. The top-down gene shaving method finds clusters of genes in which the gene expression varies greatly over the tissue samples while simultaneously maintaining a high level of coherence between the gene expression profiles; the cluster size is rigorously governed by the Gap statistic. In contrast, the EMMIX-GENE method uses the EMMIX program together with factor analyzers to first perform a filtering step and obtain a subset of relevant genes, and subsequently perform clustering of genes followed by clustering of tissues; there is no rigorous method for the choice of cluster size. Another major difference in the two methods is the way in which the clusters are constructed. An advantage of the gene shaving method is that it is totally nonparametric. In addition, the gene-shave clusters are orthogonal to each other and are of varying sizes, so that once a specific structure is captured in one cluster, the same structure will no longer be captured in subsequent clusters. However, overlapping genes are allowed between clusters if such genes induce different groupings of the columns (tissue samples). We observe that the maximum Gap statistic is useful in identifying the smallest and most coherent cluster, and thus is an excellent tool for identifying small clusters of genes. However, picking the largest cluster within a 5% or 10% of the Maximum Gap statistic is recommended in practice; this is a reasonable trade-off between coherence and a larger cluster size for exploratory purposes.

Gene shaving is also flexible in allowing the user to apply any amount of supervision required during the data analysis process. *GeneClust* is implemented with this flexibility in mind and allows interaction with the user to choose the Gap tolerance level as well as the amount of supervision. The advantage of GeneClust is its great speed in producing gene clusters. The advantage of the EMMIX-GENE method is in its accuracy of the ranking of the clusters produced. The disadvantage of this method is that it is comparatively quite slow, due to the time taken to implement the select-genes step. If a less formal approach were used to select genes, for example a *t*-test or a simple rank test, then the time required would be greatly reduced.

Both methods are treated as exploratory in this article. The parameter choices are chosen by a great amount of repetitive runs or by consulting existing literature, assessing the stability of the genes in the clusters with respect to varying parameter values. For example, with gene-shaving, the number of permutations needed and the percent shaving have been studied by Hastie et al. (2000). While with EMMIX-GENE, some simulations we performed for the Alon data set of g = 1 versus g = 2 for *t* components suggest that the 90th percentile of the null distribution of the likelihood ratio test statistic for testing a single *t* component versus a mixture of two *t* components with unrestricted variances is around 9. We have decided to act in a conservative manner by taking b_1_ = 8 to have a greater chance of not deleting a gene that may have clustering potential. The value b_2_ = 8 for the minimum cluster sizes is somewhat arbitrary. Some threshold is needed to avoid spurious clusters obtained by fitting a single *t*-component to a small number of observations that are very close together.

## Figures and Tables

**Figure 6(a).**
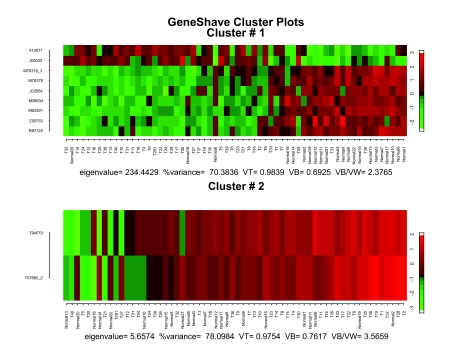
Analysis of the Alon data set (2,000 genes) under full supervision. Heat maps of the first two gene shaving clusters for the colon data with full supervision; the samples are sorted by the column mean gene.

**Figure 6(b).**
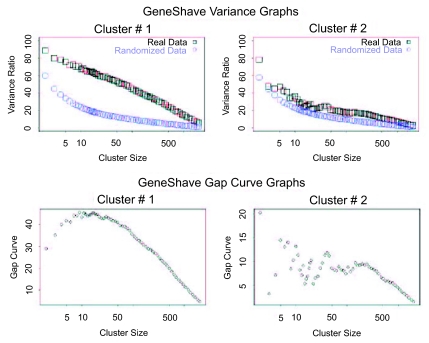
Analysis of the Alon data set (2,000 genes) under full supervision. **Top two panels:** Variance plots for the original and randomized data. The percent variance explained by each cluster, both for the original data, and for an average over twenty randomized versions. **Bottom two panels:** Gap estimates of cluster size. The Gap curve corresponds to the difference between the pair of variance curves.
